# The kinetics of metal soap crystallization in oil polymers†

**DOI:** 10.1039/d1cp03479k

**Published:** 2021-09-27

**Authors:** Joen Hermans, Lonneke Zuidgeest, Piet Iedema, Sander Woutersen, Katrien Keune

**Affiliations:** Van ‘t Hoff Institute for Molecular Sciences, University of Amsterdam PO Box 94157 1090GD Amsterdam The Netherlands j.j.hermans@uva.nl; Conservation & Science, Rijksmuseum PO Box 74888 1070DN Amsterdam The Netherlands

## Abstract

The crystallization of metal soaps in oil paint is an important chemical phenomenon that affects the appearance and structural stability of many works of art. A deep understanding of the structural transitions that occur during crystallization and their kinetics will help to support conservation decisions that minimize future detrimental change to paintings. We have used a method based on attenuated total reflection Fourier transform infrared spectroscopy and detailed spectrum analysis to quantitatively monitor all relevant metal soap structures during crystallization in a linseed oil matrix with varying degrees of polymerization. It was found that zinc soap crystallization behaviour is strongly influenced by the properties of the oil matrix, slowing down drastically with increasing polymerization, forming crystalline polymorphs in varying ratios, and demonstrating two-stage kinetics. In contrast, lead soap crystallization was invariably fast, but the degree of disorder in the crystallized phases was increasing with matrix polymerization. Besides fundamental insight into the mechanisms of metal soap crystallization, the results lay foundations for improved risk assessment during conservation treatment of oil paintings.

## Abstract 2

The kinetics of metal soap crystallization and the structures that form are highly dependent on polymer matrix properties.

## Graphical abstract

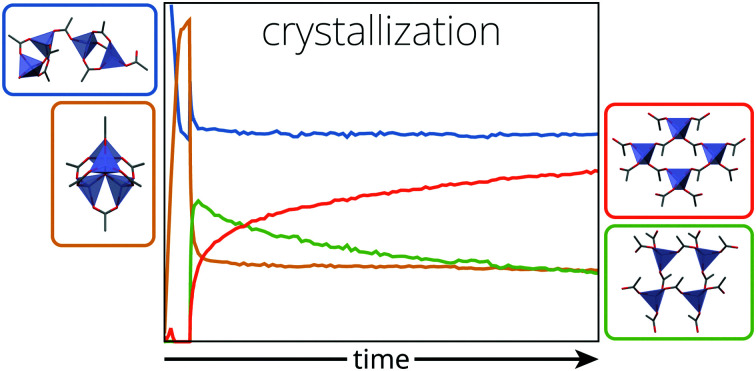

## 1 Introduction

Metal soaps are an interesting class of compounds, formed by complexation of metal ions by long-chain fatty acids. While metal soaps find application as PVC heat stabilizers,^1^ additives to ionomer or rubber formulations,^2–6^ and precursors for nanoparticle synthesis,^7^ they are also recognized as an important degradation product in historic oil paintings and other painted objects.^8,9^ In this context, metal ions from pigments or driers react with fatty acids released by the drying oil binder or paint additives, eventually forming crystalline metal soap phases that can be highly detrimental to the appearance and structural integrity of valuable works of art.^10–15^ In oil paintings, the most common metals involved in metal soap formation are lead (usually derived from hydrocerussite, minium or litharge pigments/driers) and zinc (derived from zinc oxide pigments), but calcium, copper and potassium soaps have been reported as well.^11,16,17^ Linseed oil is a common drying oil used as a paint binder that can supply saturated fatty acids for metal soap formation.

From analysis of cross-sections of historical paint samples, it is clear that there is considerable variation in the extent and morphology of metal soap crystallization in oil paint layers, as well as in the resulting crystal structure. For example, lead soaps regularly form large crystalline protrusions that can be observed to break through surface paint layers,^18^ but in some cases they remain more homogeneously distributed in paint layers, causing increased transparency that can reveal underlying paint layers.^19^ Zinc soaps, on the other hand, have been observed to crystallize in different polymorph structures in a single paint layer, depending on the depth in that layer.^20^ We wish to understand the relevant polymer matrix effects that affect the crystallization kinetics and phase behaviour of metal soaps, to gain information about the historical conditions that have acted upon a painting and ultimately inform on conservation strategies that can be employed to minimize further changes to oil paint layers.

In a previous study, we investigated the solubility of metal soaps in oil binders using differential scanning calorimetry (DSC).^21^ That work showed that nucleation of crystalline metal soap phases required greater supersaturation with increasing polymerization of the oil matrix. There was also some evidence of remaining disorder in the crystallized phases and long-term structural transformations after the initial crystallization event. More recent studies based on attenuated total reflection Fourier transform infrared (ATR-FTIR) spectroscopy demonstrated the complex evolution of zinc carboxylate structures during transition from the amorphous to crystalline state,^22^ while other researchers have studied the coordination structures of lead carboxylates in detail with nuclear magnetic resonance spectroscopy and X-ray scattering.^23,24^ In this work, we build upon these structural insights to monitor the structural transitions during crystallization of metal soaps with ATR-FTIR spectroscopy and develop a kinetic model that describes these crystallization events.

### 1.1 Coordination structure of lead and zinc carboxylates

To aid the interpretation of the kinetics of phase transitions in metal soaps, it is useful to consider the coordination structure of zinc and lead soaps and their IR spectroscopic features in some detail.

**Fig. 1:**
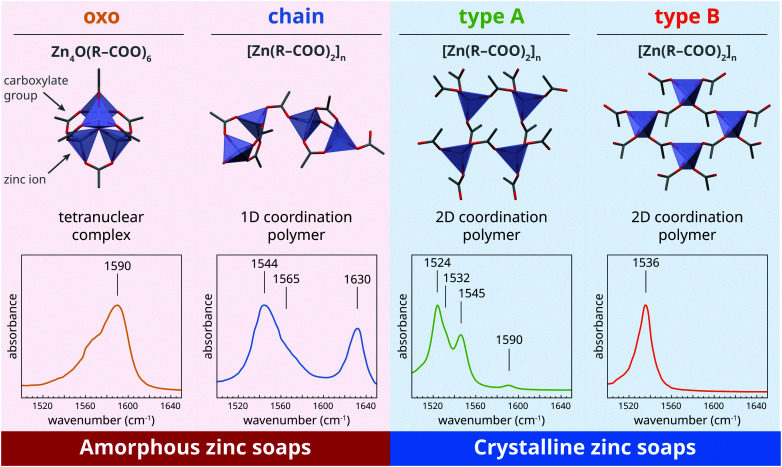
Overview of the four zinc carboxylate structures that can be adopted by zinc soaps in a polymer matrix and the corresponding features of their asymmetric carboxylate stretch vibrations bands in FTIR spectra. Carbon atoms are gray, oxygen is red, and zinc ions are indicated with blue tetrahedra. All carbon atoms except the first beyond the carboxylate group are omitted for clarity. The indicated positions of bands in IR spectra are approximate values; band positions may vary due to, for instance, disorder in coordination, variations in carboxylate backbone, and temperature. The shoulder at 1565 cm^−1^ in the spectrum of the oxo complex is considered to be caused by an impurity, possibly chain complex, given that no band shoulders have ever been reported for oxo complexes in ionomers^25,26^ or in solution.^27^ Figure adapted from ref. 22, further copyright permission related to this illustration should be directed to the ACS.

As we demonstrated previously,^22^ four distinct zinc carboxylate coordination geometries can potentially exist for zinc soaps, as shown in Fig. 1. In the amorphous phase, zinc soaps can either adopt a lineair coordination polymer ‘chain’ structure, or a tetranuclear ‘oxo’ complex that includes a O^2−^ anion in the center of the cluster. An equilibrium has been shown to exist between these two structures that is dependent on the availability of water and the concentration of carboxylic acids:^27–29^1
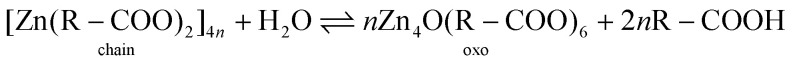
The chain and oxo complex can be easily distinguished by FTIR spectroscopy. While the highly symmetric oxo complex shows a single asymmetric carboxylate stretch vibration band centered at approximately 1590 cm^−1^, the chain complex is characterized by two main bands and a shoulder at 1544, 1630 and 1565 cm^−1^ as a result of coupling of carboxylate vibrational modes.

There are also two crystalline polymorph structures for zinc soaps (Fig. 1).^20,30^ The differences between these layered two 2D coordination polymer structures are rather subtle, with a completely symmetric orientation of ZnO_4_ tetrehedra and hexagonal alkyl chain packing in the ‘type B’ polymorph, and an alternating ZnO_4_ tetrahedra structure and square chain packing in the ‘type A’ polymorph. For zinc soaps of long-chain saturated fatty acids (*i.e.* a length of ten carbon atoms or more), the type B polymorph is the most stable structure at room temperature. Despite the subtlety of the distinction between type A and type B zinc soaps structures, their asymmetric carboxylate stretch vibrational modes are rather different, with type B zinc soaps showing a single band at 1536 cm^−1^ while type A zinc soaps give rise to four bands and a maximum at 1524 cm^−1^.

It is important to note that, in the case of zinc soaps, crystallization is not just a process of packing individual molecules or ions in a lattice, but it has characteristics of a chemical reaction. While the chain, type A and type B structures all have the same stoichiometry, crystallization from the oxo complex requires reaction with two additional fatty acid molecules and a loss of water. Moreover, the transition from either the oxo or the chain complex to one of the crystalline polymorphs requires breaking and reformation of Zn–O bonds, because each carboxylate group is bridging a different set of zinc ions in the crystalline structures. These structural details of zinc soaps make it interesting to investigate whether there are differences in crystallization kinetics for the oxo and chain complex, and whether there are conditions that favour either the type A or type B polymorph.

**Fig. 2:**
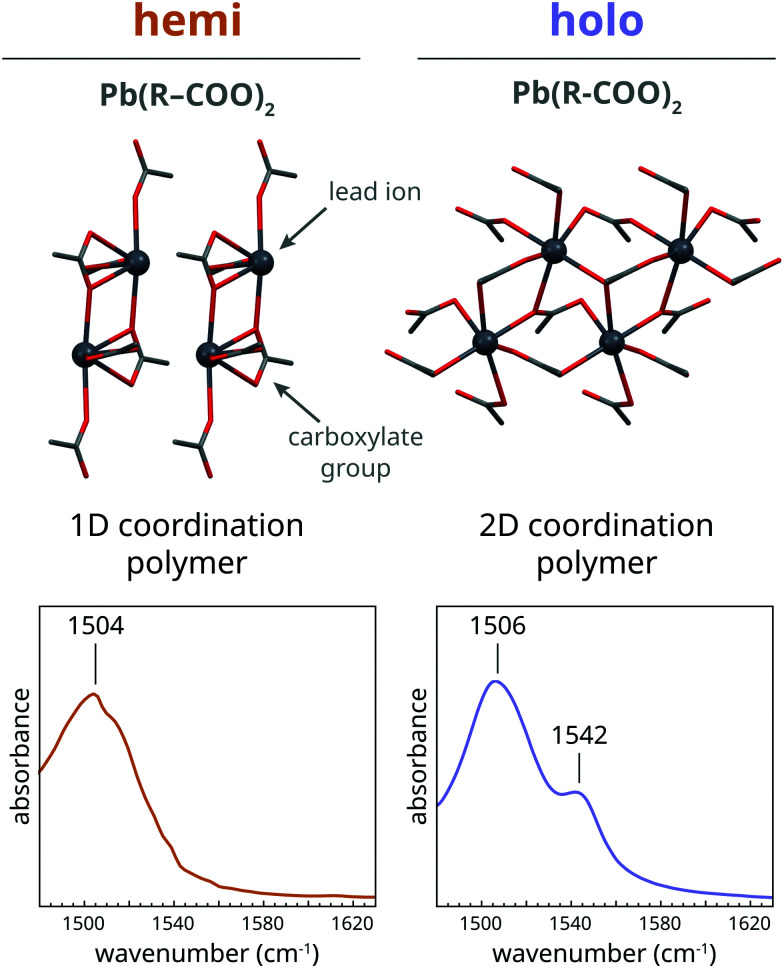
The two lead carboxylate structures adopted by crystalline lead soaps, characterized by either a hemi-directed or holo-directed coordination of lead ions, and the corresponding features of their asymmetric carboxylate stretch vibrations bands in FTIR spectra. Carbon atoms are gray, oxygen is red, and lead ions are indicated with dark grey spheres. All carbon atoms except the first beyond the carboxylate group are omitted for clarity. The indicated positions of bands in IR spectra are approximate values; band positions may vary due to, for instance, disorder in coordination, variations in carboxylate backbone, and temperature.

The landscape of possible lead soap coordination geometries is less clear than for zinc soaps. Several researchers^23,24^ have demonstrated that two distinct types of lead carboxylate coordination exist for lead soaps, as illustrated in Fig. 2. Short-chain lead soaps tend to crystallize in a ‘hemi-directed’ structure where six oxygen atoms from carboxylate groups are located on the same side of each lead ion. For long-chain lead soaps, the lead ions are more symmetrically coordinated by oxygen atoms, leading to a ‘holo-directed’ structure. In FTIR spectra, the holo structure can be distinguished from the hemi structure mainly by the appearance of a shoulder at approximately 1542 cm^−1^ next to a main asymmetric carboxylate stretch vibration band at 1504–1510 cm^−1^. In the amorphous phase, however, the details of lead carboxylate coordination are less certain, while most evidence points to a hemi structure. In the high-temperature FTIR spectrum of liquid lead soaps, a single asymmetric carboxylate stretch vibration band can be observed, though it is shifted to higher wavenumbers.^31^ The hemi structure is also favoured for shorter fatty acids, which, like in the melt, is a situation where the van der Waals interactions between fatty acid alkyl chains are reduced.^23^ Finally, Martínez-Casado and co-workers demonstrated with Pair Distribution Function (PDF) analysis that there are similarities in coordination in the liquid phase of long-chain lead soaps and the hemi structure of short-chain soaps.^24^ Combining this evidence, we hypothesize that amorphous lead soaps adopt a hemi-directed structure, but that there is a potential for variation in the Pb–O bond lengths depending on the level of crystallinity that gives rise to a shift of the asymmetric carboxylate stretch vibration band to higher frequencies.

The crystallization process for lead soaps is likely to be a more simple matter than for zinc soaps. Martínez-Casado *et al.* showed that the liquid phase of lead soaps still contains locally ordered lead carboxylate structures with a size of at least 10 Å.^24^ This existence of local order in the liquid state entails that the formation of a crystalline nucleus of lead soaps can achieved simply by bringing together several of these ordered lead soap domains, forming new lead-carboxylate bridges between domains in the process. Given these fundamental differences in nature of the crystallization process between lead and zinc soaps, it is worthwhile to compare crystallization kinetics for these two types of metal soaps and try to relate those to the morphologies of metal soap phases in oil paint systems.

### 1.2 Model system design

As a model system for metal soap crystallization in oil paint, we carried out rapid cooling experiments from the melt with mixtures of either zinc palmitate (ZnPa) or lead palmitate (PbPa) in linseed oil (LO). These initially liquid mixtures could be kept at high temperature for variable periods of time up to 120 min to induce increasing degrees of matrix polymerization due to autoxidative drying of the linseed oil.^32^ Relating the experiments to real oil paintings, this model system represents a situation where metal ions and fatty acids have already formed complexes to yield a concentration of metal soaps that is supercritical at room temperature.^21^ The increasing levels of oil polymerization in the model system relate to the differences in oil network connectivity that may arise in oil paints due to compositional variations and the degree of long-term degradation processes.

## 2 Materials and methods

### 2.1 Sample preparation

ZnPa and PbPa were synthesized by precipitation from an alkaline solution of the metal nitrate and palmitic acid, as described previously.^33,34^ The precipitated powders were thoroughly rinsed with water and acetone to wash away unreacted reagents and water, after which they were dried at room temperature. Phase purity was confirmed by ATR-FTIR spectroscopy and X-ray powder diffraction.

ZnPa–LO and PbPa–LO mixtures were prepared in a 1 : 3 weight ratio of metal soap and oil. The mixtures were ground for several minutes with a small mortar and pestle to ensure a fine dispersion of metal soap particles.

### 2.2 ATR-FTIR spectroscopy measurements

ATR-FTIR spectra were collected on a Frontier spectrometer (PerkinElmer) equipped with a heatable diamond GladiATR module (Pike Technologies). Individual spectra were collected as a single scan and 4 cm^−1^ resolution. For ZnPa crystallization measurements, the relative humidity above the sample was kept constant at 50% using a stainless steel sample cell^35^ and a home-built humidity PID-controller.

Measurement runs were carried out as follows: (1) a spectral background was collected at 25 °C; (2) the ATR top plate was heated to 150 °C; (3) a small amount of ZnPa–LO or PbPa–LO paste large enough to cover the entire ATR crystal was applied to the crystal, and automated spectrum collection was immediately started with 2–6 spectra per min, depending on the rate of crystallization; (4) the measurement cell was placed over the sample with a 50% relative humidity flow on its inlet (only for ZnPa–LO); (5) After a set time (between 1 and 120 min), the sample was quenched to 25 °C by stopping the heating module and placing heavy brass ring that was pre-cooled to −18 °C on the ATR top plate; (6) after reaching 25 °C, the temperature was kept constant, and spectrum collection was continued for up to 1000 min. A good estimate of the temperature of the sample could be obtained from spectral data by using the linear relationship between temperature and the background phonon signal of the diamond ATR crystal at 2155 cm^−1^ (Fig. S1, ESI†).

### 2.3 Data analysis

All data was processed and analyzed using custom Wolfram Mathematica scripts. A detailed description of the analysis workflow and examples of Mathematica notebook files can be found in the ESI.† A brief description of the method is given below.

#### 2.3.1 Spectrum analysis

Each spectrum in a measurement series was pre-processed by normalizing to the ester C=O stretch vibration band around 1740 cm^−1^, chopping to the region of the asymmetric carboxylate stretch vibration band, and subtracting a linear background between the edges of the spectral window. For ZnPa, a hybrid non-linear spectral fit was carried with fixed reference spectra for the crystalline ZnPa species and variable sets of Gaussian bands for non-crystalline structures.^22^ Strong constraints were imposed on the positions, widths and relative intensities of the Gaussian bands to ensure meaningful results. For PbPa, the spectra were modeled as a linear combination of the first and the last spectrum in a series, setting *t* = 0 on the last spectrum before crystallization starts.

#### 2.3.2 Kinetic analysis

The fractional concentration curves that were produced by fitting of the ATR-FTIR spectra were fit with kinetic models described in the Results section. For ZnPa, the three curves describing the concentrations of oxo, chain and crystalline ZnPa structures were fit simultaneously with a global parameter optimization algorithm, applying constraints on the fit parameters to obtain reasonable results. For PbPa, a simple exponential model was fit to the concentration curves of crystalline PbPa without constraints.

## 3 Results

The crystallization behaviour of ZnPa and PbPa will be discussed in succession. For each metal soap, we will first consider the relevant structural transitions, and then proceed to characterize the kinetics of these transformations.

### 3.1 Structural transitions during ZnPa crystallization

**Fig. 3:**
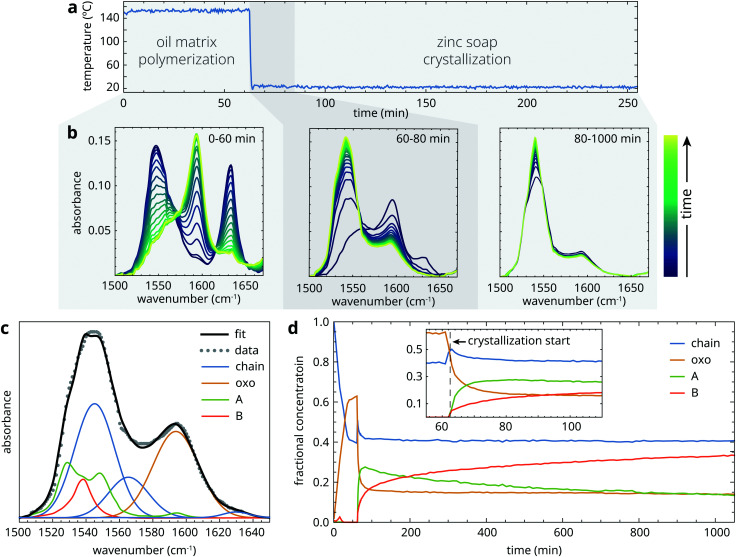
Overview of collected data during a ZnPa crystallization experiment with 60 minutes polymerization time. (a) Temperature of the sample during first 250 minutes of the experiment. (b) Three series of ATR-FTIR spectra that illustrate the dominant zinc carboxylate transformation processes in each time domain. (c) Example of a spectral fit to asymmetric carboxylate stretch band envelope. (d) Fractional concentration profiles of the four zinc carboxylate structures during the crystallization experiment.

Prior to heating, samples started out as a suspension of crystalline zinc palmitate (type B structure) in liquid linseed oil. The crystallization experiments can be divided in three phases, as illustrated in Fig. 3a: (1) a heating phase at 150 °C of variable duration, during which the sample is a homogeneous liquid that gradually thickens due to oil autoxidation; (2) a fast crystallization phase (*t* ≈ 0.5–30 min) immediately after quenching to 25 °C during which the sample mixture becomes supersaturated, leading to ZnPa precipitation; (3) a long-term (re-)crystallization phase on a timescale of hours to days, during which there can be further crystal growth or polymorph transitions. To investigate the structural transitions in ZnPa crystallization experiments in more detail, we take the experiment with 60 minutes heating time as an example. Typical spectral changes in the asymmetric carboxylate stretch band envelope corresponding to each of the time domains are shown in Fig. 3b.

Initially, ZnPa adopted the chain complex structure in the liquid phase at 150 °C. However, a partial conversion process from chain to oxo complex started immediately, tailing off at a conversion of approximately 50%. In most cases of chain-oxo conversion reported in the literature, this transition is driven by the presence of water, following reaction (1).^27,29^ However, in this experiment it is suspected that water does not drive the chain to oxo complex conversion, because the water concentration in an oil matrix at 150 °C is extremely low. It is likely that an oxygen species capable of supplying the oxygen anion in the oxo complex is generated during oil autoxidation. The fact that the chain to oxo complex conversion was incomplete suggests that there is an equilibrium between the two zinc carboxylate species that relies on the carboxylic acid concentration and the unknown oxygen species.^22^ In what follows, we will not consider this chain to oxo complex conversion and its kinetics further. Apart from the experiments with the shortest heating times (*t* < 20 min), the starting concentrations of chain and oxo complex were comparable for each experiment.

Upon quenching to 25 °C (which took approximately 1 minute), many changes to the structure of zinc palmitate occurred simultaneously. Firstly, the decreasing temperature slightly shifted the equilibrium between the chain and oxo complex in favour of the chain complex. Just after the sample reached 25 °C, the characteristic carboxylate features of crystalline ZnPa grew at the expense of the chain and oxo complex in the ATIR-FTIR spectra (Fig. 3b). While both type A and type B polymorphs formed immediately, the concentration of the metastable type A polymorph was initially higher (Fig. 3d). Interestingly, the initial crystallization process was incomplete. Approximately 1 h after quenching, the concentration of non-crystalline ZnPa changed only very slowly, while only approximately 40% of the total concentration of ZnPa was in a crystalline state. There was also a stark difference in crystallization behaviour between the chain and oxo complex. At the start of crystallization, their concentrations were roughly equal. After the initial crystallization phase, however, the chain complex had only decreased by 20% of its initial concentration, while the oxo complex had decreased by 70%.

After the initial crystallization process, which lasted approximately 20 minutes after a heating time period of 60 minutes, the system of ZnPa in linseed oil remained reactive. Fig. 3b and d show that there was a polymorph transition occurring from type A to type B in ZnPa. At the end of the measurement run, 1000 minutes after quenching, all four zinc carboxylate coordination structures were still present. However, both the crystallization process and the polymorph transition continued on a longer timescale. When a comparable ZnPa in oil sample that was oven-heated at 150 °C for 60 minutes was analyzed with ATR-FTIR spectroscopy again after 3 days, no amorphous ZnPa could be detected anymore (Fig. S2, ESI†). A further 49 days after heating, there was almost exclusively type B ZnPa present in the sample. These observations demonstrate that, while there is a fast crystallization process in partially polymerized oil, a fraction of the zinc soaps is characterized by very slow crystallization kinetics.

### 3.2 Oil matrix influence on ZnPa crystallization

Having identified the changes in the structure of zinc soaps that occur during crystallization, we can investigate how the crystallization process depends on the properties of the surrounding matrix. As a control experiment, we observed that a sample of pure liquid ZnPa crystallized completely and directly in a type B structure in approximately 30 s (Fig. S3, ESI†).

**Fig. 4:**
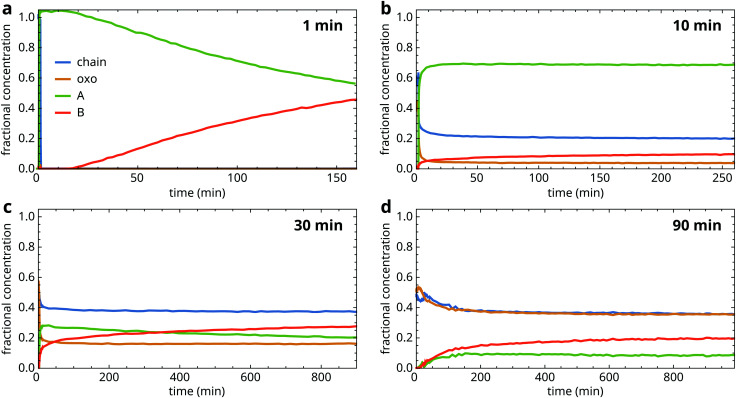
Fractional concentration profiles of the chain, oxo, type A and type B ZnPa species during crystallization in a polymerizable oil matrix at (a) 1 min, (b) 10 min, (c) 30 min and (d) 90 min heating time at 150 °C. Time *t* = 0 is set at the start of quenching.

Fig. 4 shows fractional concentration curves for the four types of zinc carboxylate structure for several durations of heat treatment at 150 °C. These experiments illustrate the different types of behaviour during ZnPa crystallization in an oil matrix. Interestingly, the oil matrix has an influence on ZnPa crystallization even when it is unpolymerized. When ZnPa/oil samples were heated for only 1 min, just long enough to achieve a homogeneous liquid mixture, ZnPa crystallized completely within 30 s in the type A polymorph rather than type B (Fig. 4a). However, a polymorph transition occurred on a much slower timescale, eventually leading to the formation of the more stable type B polymorph.

At 10 minutes heating time, the crystallization process looked strikingly different (Fig. 4b). While the initial crystallization was still rapid, it was no longer complete. Approximately 30% of ZnPa remained non-crystalline at 250 minutes. Moreover, ZnPa persisted in its type A form for much longer, with only a 10% fraction of type B ZnPa detected at the end of the run.

At 30 minutes, initial crystallization started to slow down and the fraction of ZnPa that was characterized by very slow crystallization kinetics further increased (Fig. 4c). Notably, at higher degrees of polymerization the fraction of ZnPa that crystallized directly into the type B polymorph increased. Fig. 4d demonstrates that this trend continued, with type B ZnPa representing the majority of crystalline ZnPa at all times during the experiment at 90 min heating time. In this more highly polymerized system, only approximately 20% of ZnPa crystallized after 17 hours.

### 3.3 Kinetic model for ZnPa crystallization

**Fig. 5:**
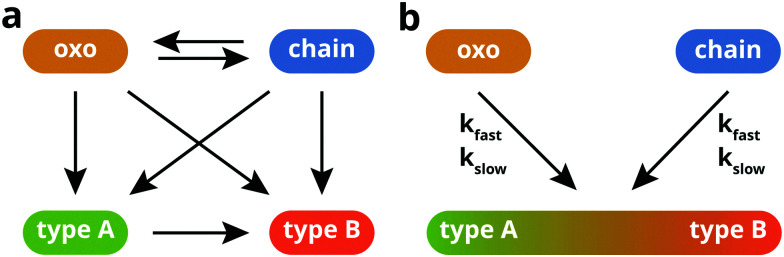
Illustrations of (a) all possible reactions that can occur during ZnPa crystallization, and (b) the reactions that were considered in the simplified kinetic model.

To gain more insight into the kinetics of the individual processes occurring during zinc soap crystallization, we constructed a kinetic model to describe the concentrations of ZnPa species over time. In theory, there are seven possible reactions between the different ZnPa species, as illustrated in Fig. 5a. It is known that there exists a (partial) equilibrium between the oxo and chain complex, both these non-crystalline species can theoretically crystallize to either a type A or B polymorph structure, and finally Fig. 4 clearly shows that there is a type A to type B polymorph transition. The spectroscopic data also suggests that at least some of these reactions have a fast and a slow component. For a kinetic model, this reaction scheme needs to be simplified, because it includes multiple reaction paths leading to the same product that are difficult if not impossible to distinguish spectroscopically (*e.g.* type B ZnPa can be formed from the oxo complex directly, *via* the chain complex, or *via* a type A polymorph), Moreover, the complete reaction scheme would lead to an underdetermined system of equations.

The simplified model, illustrated in Fig. 5b, serves to allow a more quantitative comparison between ZnPa crystallization at increasing levels of matrix polymerization. For this model, we made several assumptions and approximations. Firstly, we assumed that there is no transition between the chain to oxo complex during crystallization. This transition is likely to have little effect on the overall kinetics of the system, because the factors that affect oxo-chain equilibrium (carboxylate and water concentrations, and temperature) are all approximately constant from the moment crystallization starts, and the rate constants of oxo and chain consumption are similar. Secondly, we neglected the distinction between type A and type B polymorphs, grouping their contributions as a single concentration of crystalline ZnPa species. While it could be the case that type A and type B ZnPa form in different ratios in the course of a crystallization experiment, this assumption entails that the rate constants for the formation of type A or type B are the same. Finally, while the crystallization from the oxo complex consumes two palmitate molecules and releases a molecule of water, these reactants are not included explicitly in the model. This omission entails the assumption that palmitate or water molecules are not involved in the rate-determining step of the crystallization process.

Having limited the relevant crystallization reactions to transitions from either oxo or chain to crystalline ZnPa (Fig. 5b), we can now write a set of three differential equations that describe the rate of change in concentration of each ZnPa species. It was found that the shape of the concentration curves was described much better across the range of heating times by second-order reaction kinetics (Fig. S4, ESI†). Incorporating a fast and slow kinetic component for the two crystallization reactions, the solution of the set of differential equations gives the fractional concentrations of each of the three considered structures over time:2
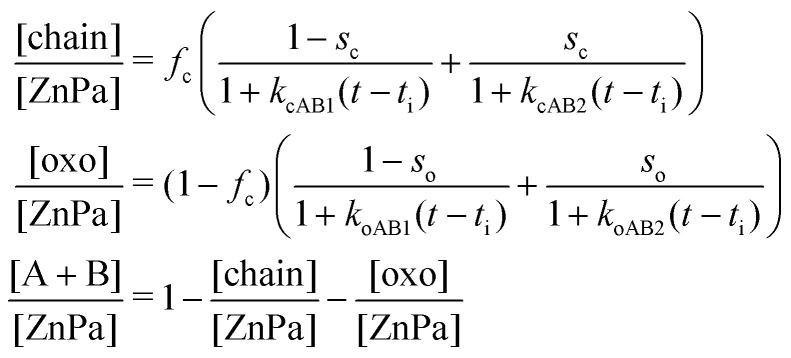
where *t*_i_ is an initiation time, *f*_c_ is the fraction of non-crystalline ZnPa in the chain complex structure prior to crystallization and [ZnPa] is the total concentration of ZnPa. *k*_cAB1_, *k*_cAB2_ and *s*_c_ describe the kinetic behaviour of the chain complex, and they are the fast and slow rate constants for crystallization, and the fraction of chain complex that is characterized by slow kinetics, respectively. *k*_oAB1_, *k*_oAB2_ and *s*_o_ are the equivalent parameters for the oxo complex. This set of equations was fit to the corresponding set of experimental concentration profiles using a global parameter optimization algorithm and constraints on the fit parameters to meaningful values (see ESI† for details of the fit procedure).

**Fig. 6:**
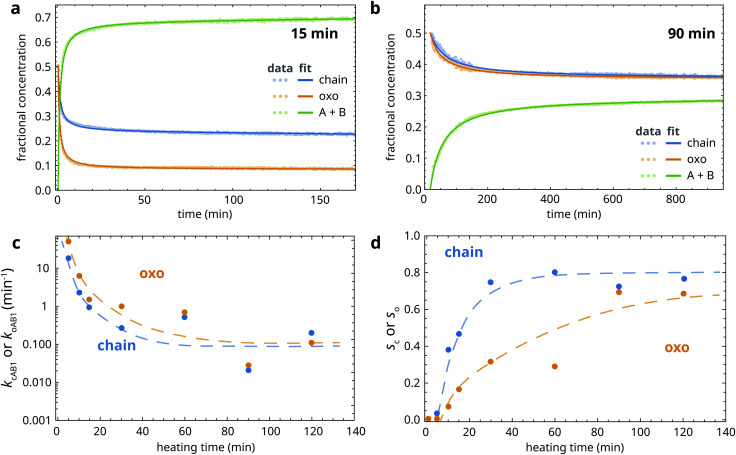
Fit of the model in eqn (2) to the experimental data corresponding to (a) 15 min and (b) 90 min heating time. (c) Rate constants *k*_cAB1_ and *k*_oAB1_ as function of heating time. (d) Slow fractions *s*_c_ and *s*_o_ as function of heating time. All dashed curves are guides to the eye.

The simplified kinetic model gave excellent fit results for all experimental runs. Fig. 6a and b show examples of a fit of eqn (2) to concentration profiles corresponding to 15 and 90 min heating time. The crystallization of ZnPa at 1 min heating time was complete within the time necessary to collect a spectrum, so the kinetic fit was not considered meaningful for that run.

Despite the limitations of the simplified two-reaction model of Fig. 5b, the kinetic fit yields some interesting observations. As was observed qualitatively before, crystallization became slower and the initiation time increased with increasing polymerization of the oil matrix. Fig. 6c shows that the fast-component crystallization rate constants decreased by more than two orders of magnitude beyond one hour polymerization time. At short heating times, the crystallization rate of oxo complex was consistently higher than the chain complex. However, at heating times of 60 minutes and longer, the rates of crystallization became comparable for both types of amorphous ZnPa species. There was considerable uncertainty in the values of the slow-component rate constants, since the slope of the concentration curves at longer timescales was very minimal. However, the slow-component rate constants were usually 10^4^–10^5^ times slower than the fast rate constants.

There were also clear differences between the two complexes in the fraction of zinc carboxylates characterized by slow and fast kinetics, as shown in Fig. 6d. At all heating times where both amorphous structures were present before quenching, the oxo complex crystallized to a greater extent in the initial fast crystallization phase.

While the type A to type B polymorph transition could not be included in the kinetic model, it was possible to investigate the transition qualitatively. Fig. S5 (ESI†) shows the fraction of crystalline ZnPa in the type A polymorph structure over time. Irrespective of heating time, all samples exhibited a slow polymorph transition to type B. Interestingly, all curves except for the 1 min heated sample have a very similar slope, which suggests that the rate of the polymorph transition is largely independent of the degree of polymerization in the matrix.

### 3.4 Structural transitions during PbPa crystallization

**Fig. 7:**
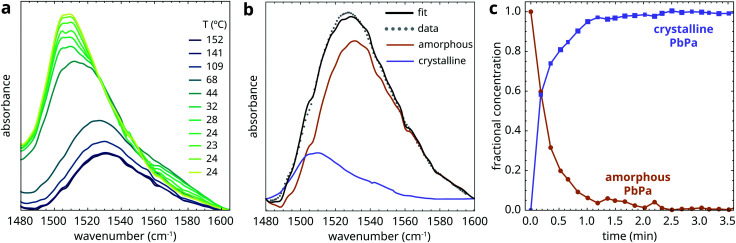
(a) ATR-FTIR spectra showing the asymmetric carboxylate stretch vibration band of a mixture of PbPa and LO during quenching from 150 °C after 30 min heating. Spectra were collected every 10 s. (b) Illustration of a linear combination fit of a spectrum collected during quenching, using the spectra at the onset of crystallization and at equilibrium as pure components. (c) Fractional concentration curves of amorphous and crystalline PbPa.

Having studied the complexity of ZnPa crystallization in detail, it is interesting to make a comparison with PbPa crystallization. Fig. 7a shows the evolution of the asymmetric carboxylate stretch vibration band of PbPa during rapid cooling to room temperature. In the course of crystallization, the band with a maximum at 1530 cm^−1^ that corresponds to amorphous PbPa is replaced by a band positioned at approximately 1508 cm^−1^. We could not detect any signs of intermediate PbPa species between the starting amorphous complex and the crystalline product by applying singular value decomposition to spectral series acquired during crystallization. While Martínez-Casado and co-workers report the existence of a rotator phase between the melt and crystalline PbPa,^31^ this phase is likely to be either too short-lived and/or not spectroscopically distinct enough to be detected in our experiments.

The results indicate that the spectra collected during crystallization of PbPa can be modeled simply as a linear combination of the spectrum at the onset of crystallization and the final spectrum in the series at equilibrium. Fig. 7b illustrates an example of such a fit, and Fig. 7c shows the resulting fractional concentration curves of amorphous and crystalline PbPa. This approach using two-component linear combination fitting and pure component spectra sourced from the dataset itself gave very good fits to experimental spectra for all studied samples (*R*^2^ > 0.997).

### 3.5 Oil matrix influence on PbPa crystallization

**Fig. 8:**
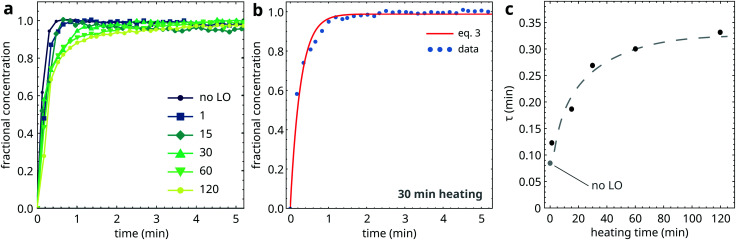
(a) Fractional concentration curves of crystalline PbPa, and a comparison with pure PbPa without a LO matrix (no LO). In these curves, *t* = 0 is set at the onset of crystallization. (b) Example of a fit to eqn (3) of the PbPa concentration curve for 30 min heating time. (c) Crystallization timescales *τ* as function of heating time at 150 °C. The dashed curve is a guide to the eye.

Mixtures of PbPa and LO were subjected to increasing durations of heating at 150 °C to induce oil polymerization, after which the mixtures were quenched to 25 °C. Fig. 8a shows the fractional concentration of crystalline PbPa for heating times ranging from 1 to 120 min. For comparison, a crystallization curve corresponding to pure PbPa without LO is also included. From these curves, it is clear that PbPa crystallization is invariably fast and that the degree of matrix polymerization has only limited influence on the crystallization kinetics of PbPa. In all samples, crystallization was more than 90% complete within 2 min after the start of crystallization.

The high crystallization rate of PbPa makes it challenging to select an adequate kinetic model for the entire series of experiments. For the more polymerized systems, a second-order model seemed to describe the crystallization process best (Fig. S6, ESI†). However, for pure PbPa and PbPa–LO mixtures at heating times of 1 and 15 min, the crystallization process was already complete after the collection of three spectra. Such a small number of data points inevitably leads to large uncertainties in the kinetic rate constants and the shape of the crystallization curve. We chose to compare the timescales of crystallization instead, by fitting the concentration curves with a simple exponential model:3
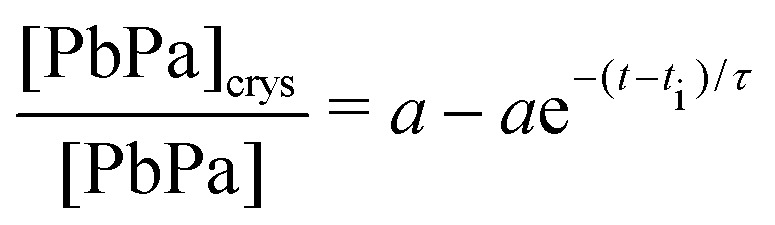
where [PbPa]_crys_/[PbPa] is the fractional concentration of crystalline PbPa, *τ* is the timescale of crystallization, *t*_*i*_ is an initiation time, and *a* is the equilibrium concentration of crystalline PbPa with a value very close to 1. Fig. 8b shows an example of a fit of this model to the crystallization curve corresponding to 30 min heating time. The model gave good fits to all data (*R*^2^ > 0.999). The crystallization timescales clearly increased with increasing oil polymerization (Fig. 8c). However, the timescale increased only by approximately a factor of 3 compared to pure PbPa, and the value showed only minor changes after 30 min heating.

**Fig. 9:**
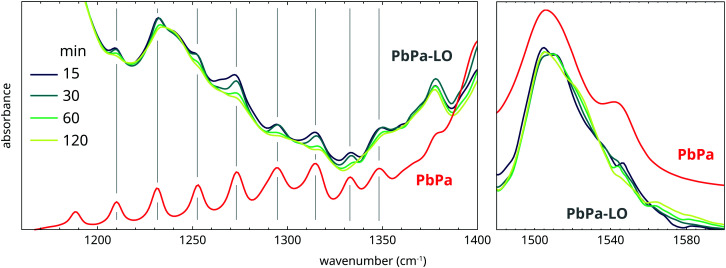
Comparison of normalized ATR-FTIR spectra of increasingly polymerized mixtures of PbPa and LO, and pure PbPa. Spectra were collected several minutes after quenching from 150 °C.

While the properties of the oil matrix do not seem to strongly affect PbPa crystallization kinetics, it is interesting to consider the structure of the crystalline PbPa phases by studying the ATR-FTIR spectra of the mixtures after crystallization. Fig. 9 compares the spectra collected several minutes after quenching for increasingly polymerized PbPa–LO mixtures and for pure PbPa. The progression bands between 1150 and 1370 cm^−1^ are caused by coupling of the wagging vibrations of the CH_2_ units in the alkyl chains of palmitate chains.^34^ These bands only appear when the alkyl chains are packed in an all-*trans* conformation. For PbPa crystallized in LO, the intensity of the progression bands clearly decreased with increasing heating time, indicating that the polymer matrix induces conformational disorder in the crystallized PbPa phases. This observation is in line with previous research, which demonstrated that the crystallization enthalpy of lead soaps decreased with oil matrix polymerization.^21^ Additionally, the asymmetric carboxylate stretch vibration band shows no clear shoulder at 1540 cm^−1^ for PbPa crystallized in LO, though there are some signs of either band broadening or a shoulder at approximately 1525 cm^−1^. Together, these spectroscopic observations suggest that while the crystallization rate is not strongly affected by the polymer matrix, the oil environment does cause disorder in the alkyl chain packing, which in turn induces the adoption of a hemi-directed carboxylate coordination around the lead ions. It is not clear if or on what timescale this structure converts to the more stable holo-directed conformation.

## 4 Discussion

The time-dependent ATR-FTIR experiments have provided many insights into the mechanisms and kinetics of metal soap crystallization. It is important to note that these quenching experiments reflect a situation where a relatively high concentration of metal soaps (25% by weight) is already present prior to crystallization. In oil paintings, metal soap crystallization may start before such high concentrations can build up in paint layers. In that case, the crystallization process is likely to be slower due to a lower supersaturation of metal soaps. The experiments reported here were designed to study the effects of polymerization of the oil matrix surrounding the metal soaps, and the observed effects are likely to be relevant across a larger range of metal soap concentrations.

The observation that ZnPa, and possibly also PbPa, crystallization in oil is best described with second-order kinetics provides some more understanding of metal soap crystallization. We can envision that the crystallization of metal soaps from the liquid state is composed of two processes: an internal structural rearrangement (packing of the fatty acid chains and induced changes in metal carboxylate coordination), and a diffusion process (the movement of metal soap ‘units’ to form or join a crystalline nucleus). While the internal rearrangement is a first-order process, second-order kinetics would be expected if diffusion is the rate-limiting step during metal soap crystallization. The second-order shapes of the crystallization curves for ZnPa in Fig. 6, and potentially also for PbPa in Fig. S6 (ESI†), suggest that diffusion is indeed the key process that limits crystallization rate for metal soaps in a polymerized matrix.

Focusing on ZnPa, it is interesting that the initial crystallization process was incomplete in polymerized oil. The existence of a fraction of amorphous ZnPa with crystallization kinetics orders of magnitude slower than the primary crystallization process suggests that there is variation in the local environment of non-crystalline ZnPa. Perhaps a fraction of ZnPa in the chain and oxo geometry is tangled in relatively densely polymerized pockets of the oil matrix, making the necessary structural rearrangement for the formation of type A or type B crystalline nuclei much slower. If this is true, it could even be that the formation of new nuclei is practically impossible for this fraction of ZnPa, and that this fraction contributes to crystallization exclusively through a slow process of dissolution and growth onto existing crystalline phases. This link between matrix polymerization and crystallization mechanism is supported by the observation that the fraction of ZnPa characterized by slow kinetics increased with the degree of polymerization (Fig. 6d), and it is in line with previous observations on the concentrations of chain and oxo species in paint films that pointed to the existence of variation in local conditions in oil polymers.^22^ The fact that a larger fraction of the chain complex is characterized by slow kinetics than oxo and therefore more susceptible to matrix polymerization (Fig. 6d) suggests that either crystallization from chain complex requires more molecular rearrangement, or that the chain complex is more likely to reside in polymer environments that force slower crystallization.

Generally, the crystallization rate of the oxo complex was slightly faster than the chain complex (Fig. 6c). This observation seems to contradict previously published results, where we found evidence that oxo complexes are consumed more slowly than the chain complex.^22^ However, in those earlier experiments based on mixtures of ZnO and LO, ionomeric oxo and chain complexes needed to react with *in situ* generated saturated fatty acids prior to crystallization, while the ZnPa complex has already formed in the experiments described here. This distinction could be important. Combining the current and previous observations, the results suggest that the zinc soaps in the oxo geometry may crystallize slightly faster, but that ionomeric chain complexes exchange polymer network-bound carboxylate groups for those of fatty acids more easily. Finally, it should be noted that, so far, the differences in reactivity between chain and oxo complexes have proven to be rather limited compared to all other factors that influence the kinetics of metal soap formation.

The reported experiments also shed light on the relationship between ZnPa crystallization kinetics and crystalline polymorph structure. While pure ZnPa crystallizes immediately into the stable type B polymorph (Fig. S3, ESI†), the presence of a liquid oil matrix causes the initial adoption of a type A structure. This effect is probably due to a disruption of optimal packing of the palmitate chains by the oil matrix, which in turn causes ZnPa to adopt the type A structure that is characterized by more favourable carboxylate coordination around zinc.^20^ Interestingly, as oil matrix polymerization starts to slow down crystallization, type B ZnPa becomes the more dominant polymorph again during the initial crystallization phase. These observations have important implications for oil paintings research. The persistence of type A polymorphs for zinc palmitate or stearate in real oil paint samples^20^ can now be associated with a fast and possibly also recent crystallization process, perhaps caused by a sudden change in climate conditions (high temperature or relative humidity) or certain conservation procedures (solvent/water cleaning or consolidation treatments). As such, zinc soap polymorph detection can give information on the conservation history of a painting.

Comparing the results of PbPa and ZnPa, PbPa crystallization was markedly faster than ZnPa crystallization, especially at higher degrees of polymerization of the oil matrix. This result is in line with the fact that there is much more structural similarity between the liquid and crystalline state in lead soaps than in zinc soaps, as discussed in the Section 1.1. The presence of more local order in the liquid state of PbPa could mean that liquid PbPa essentially already contains nuclei for crystallization, explaining why crystallization was relatively independent of matrix polymerization for PbPa. In contrast, for nucleation of crystalline ZnPa phases, bonds need to be broken and substantial molecular rearrangement needs to take place.

It could be said that crystallization of lead soaps is both more simple and more complex than in the case of zinc soaps. On the one hand, PbPa crystallization could be adequately described as a simple *X* → *Y* transition running to completion, and the effects of the polymer matrix on the crystallization process were far more limited than for ZnPa. On the other hand, there seems to be more room for varying degrees of conformational disorder in PbPa, while the coordination geometries of ZnPa are mostly limited to four well-defined ZnPa species (possibly with the exception of disorder in the conformation of the chain complex^29^). As a consequence, while the kinetics of crystallization remained similar, the degree of polymerization of the oil matrix did influence the level of order of the PbPa phases that crystallized. For oil paintings, these varying degrees of order in the lead soap phases could influence the rate of further chemical processes that involve lead soaps, for instance recrystallization to other lead salts^36^ or lead ion migration. It is also interesting to attempt to link these differences between the metal soaps to the metal soap morphologies observed in oil paint systems. The commonly observed lead soap protrusions with dimensions of hundreds of micrometers suggest there is a process similar to Ostwald ripening at play, where larger crystalline PbPa phases grow at the expense of smaller ones. For such a process to occur, there needs to be an equilibrium between lead soap crystallization and dissolution, and a non-zero diffusion coefficient to allow movement of lead ions and fatty acids between crystalline phases. With the results reported here, we hypothesize that the relatively disordered nature of lead soap phases in oil paint polymers makes it easier for precipitated lead soap structures to dissolve and reform elsewhere. In comparison, zinc soaps were shown to have a lower solubility in oil and a higher activation energy for crystallization,^21^ making it more likely that they can remain dispersed as small crystallites for long periods of time after precipitation.

Given the differences in the nature of the crystallization process for lead and zinc soaps, it is also likely that they are not affected in the same way by environmental factors that may influence metal soap crystallization, such as the moisture content in paint films or the exposure to heat and organic solvents. Experiments to study these effects are the topic of a future publication.

## 5 Conclusions

ATR-FTIR spectroscopy can give highly detailed insight into the structural transitions of metal soaps during crystallization and the kinetics of these processes. With prior knowledge of the spectroscopic features of all relevant metal carboxylate coordination geometries, quantitative information on the concentrations of species can be obtained from ATR-FTIR data and kinetic models can be applied to study the mechanisms of crystallization.

Zinc palmitate crystallization is strongly influenced by the properties of the surrounding oil matrix. As the matrix becomes more polymerized, crystallization slows down dramatically and an increasing fraction of zinc soaps exhibits ultra-slow crystallization kinetics. Moreover, a metastable type A polymorph structure is formed as an intermediate at low degrees of matrix polymerization. Crystallization kinetics for zinc soaps in oil appear to be second-order, which suggests that the process is diffusion-limited under these conditions.

Lead palmitate crystallizes faster than zinc palmitate, and the kinetics are only slightly affected by matrix polymerization. However, there seem to be remaining levels of disorder in fatty acid chain packing in the crystalline lead soap phases when crystallization occurs in a strongly polymerized matrix.

The results demonstrate the ability of FTIR spectroscopy to give information on the conservation history of oil paintings and their potential for further chemical reactivity. In turn, such information can help to support restoration and conservation decisions for painted artworks.

## Author contributions

J. H.: conceptualization, formal analysis, investigation, methodology, visualization, writing – original draft, supervision, funding acquisition. L. Z.: investigation. P. I.: writing – review & editing. S. W.: writing – review & editing. K. K.: writing – review & editing.

## Conflicts of interest

There are no conflicts to declare.

## Supplementary Material

CP-023-D1CP03479K-s001

CP-023-D1CP03479K-s002

CP-023-D1CP03479K-s003

CP-023-D1CP03479K-s004

CP-023-D1CP03479K-s005
