# Supplementary material for: The kinetics of metal soap crystallization in oil polymers
Source: Phys Chem Chem Phys. 2021 Sep 27;23(39):22589–600. doi: 10.1039/d1cp03479k (PMC8514046; doi:10.1039/d1cp03479k)
Supplement: CP-023-D1CP03479K-s005 [file CP-023-D1CP03479K-s005.pdf]

Electronic Supplementary Material (ESI) for Physical Chemistry Chemical Physics.
This journal is © the Owner Societies 2021

Electronic Supplementary Information

The kinetics of metal soap crystallization and polymorphtransition in oil polymers

Joen Hermans, Lonneke Zuidgeest, Piet Iedema, Sander Woutersen and Katrien Keune

*Van 't Hoff Institute for Molecular Sciences, University of Amsterdam,
PO box 94157, 1090GD Amsterdam, The Netherlands.
Tel: +31 (0)20 525 6626; E-mail: j.j.hermans@uva.nl*

Contents

1 Data analysis **2**
1.1 Spectrum analysis 2
1.2 Kinetic analysis 2

2 Linear correlation between diamond phonon signal and temperature **4**

3 Long-term ZnPa crystallization processes in LO **5**

4 ATR-FTIR spectra of pure ZnPa crystallization **6**

5 Comparison of first and second order kinetics for ZnPa crystallization **7**

6 Kinetics of crystalline polymorph conversion of ZnPa in LO **8**

7 Comparison of first and second order kinetics for PbPa crystallization **9**

1 Data analysis

All data was processed and analyzed using custom Wolfram Mathematica scripts. Example Mathematica notebook files showing details of the implementation of data processing methods can be downloaded from the article webpage.

1.1 Spectrum analysis

Spectrum pre-processing was the same for ZnPa-LO and PbPa-LO experiments. The following steps were executed for each spectrum in a measurement series:

- spectra were normalized to the ester C=O stretch vibration band around 1740 cm^{-1} to account for variations in sample contact between samples or within a series.
- spectra were chopped to the region of the asymmetric carboxylate stretch vibration band (1500–1650 cm^{-1} for ZnPa, and 1480–1600 cm^{-1} for PbPa).
- a linear background was subtracted between the edges of the spectral window (*only for ZnPa*).
- the chopped spectra were normalized to the total area of the entire envelope, to remove the effect of temperature on the absolute intensity of the carboxylate band envelope (*only for ZnPa*).

After pre-processing, the extracted and corrected carboxylate band envelopes were fit to obtain fractional concentration curves for each non-crystalline and crystalline metal soap species.

ZnPa: A hybrid non-linear spectral fit was performed with the `NonlinearModelFit` function in Mathematica, where the carboxylate band envelope was modeled as a sum of pure reference spectra for type A and type B ZnPa and a set of Gaussian bands for oxo (1 band) and chain complexes (3 bands).[1] Constraints were imposed on the Gaussian band parameters. The position and width of each Gaussian band was allowed to vary slightly around the typical value for each parameter, and the relative magnitude of the two largest bands of the chain complex spectrum was limited to a small window. All fits converged well, and for each series, at least 10 fits at various points of the measurement run were inspected manually to ensure the absence of fitting artefacts.

PbPa: Singular value decomposition of each spectral series was applied to confirm that the crystallization process was a clear A→B transition without any intermediates. After setting $t = 0$ on the last spectrum before crystallization started, the spectra were modeled as a linear combination of the spectrum at $t = 0$ and the last spectrum in a series. All fits converged well, and for each series, at least 5 fits at various points of the measurement run were inspected manually to ensure the absence of fitting artefacts.

1.2 Kinetic analysis

The fractional concentration curves that were produced by fitting of the ATR-FTIR spectra were analyzed further by the application of kinetic models.

ZnPa: the three curves describing the concentrations of oxo, chain and crystalline ZnPa (sum of type A and type B structures) were fit simultaneously with a single set of fit parameters using the global parameter optimization function `MultiNonlinearModelFit` in Mathematica, and the model described in more detail in the main text:

$$\frac{[\text{chain}]}{[\text{ZnPa}]} = f_c \left(\frac{1 - s_c}{1 + k_{cAB1}(t - t_i)} + \frac{s_c}{1 + k_{cAB2}(t - t_i)} \right) \tag{1}$$

$$\frac{[\text{oxo}]}{[\text{ZnPa}]} = (1 - f_c) \left(\frac{1 - s_o}{1 + k_{oAB1}(t - t_i)} + \frac{s_o}{1 + k_{oAB2}(t - t_i)} \right) \tag{2}$$

$$\frac{[\text{A+B}]}{[\text{ZnPa}]} = 1 - \frac{[\text{chain}]}{[\text{ZnPa}]} - \frac{[\text{oxo}]}{[\text{ZnPa}]} \tag{3}$$

[1] This approach is adaption of the methods previously published in Beerse et al. (2020), *ACS Applied Polymer Materials* 2, 12, 5674–5685.

All data points before the onset of crystallization were not taken into account for the fit. Without constraints on the model parameters, the optimization algorithm had trouble converging to global minimum solutions. To obtain reasonable results we employed the following constraints:

- k_{cAB1} and k_{oAB1} had to be in a similar order of magnitude as manually estimated values.
- k_{cAB1} and k_{oAB1} were forced to be in the same order of magnitude.
- $k_{\mathrm{cAB1}}/100 > k_{\mathrm{cAB2}} \geq 0$ and $k_{\mathrm{cAB1}}/100 > k_{\mathrm{cAB2}} \geq 0$.
- $t_{\mathrm{i}} > 0$.
- $1 > s_{\mathrm{c}} > 0$ and $1 > s_{\mathrm{o}} > 0$,
- f_{c} and f_{o} had to be very close to the experimental data points for the fractional chain and oxo concentration at the onset of crystallization.
- $f_{\mathrm{c}} + f_{\mathrm{o}} \equiv 1$

All these constraints are either dictated by logic or follow directly from observations of the data. Apart from the measurements at 1 min heating time at 150 °C, where crystallization was too fast to be modeled, all runs were described excellently by the model.

PbPa: a simple exponential model

$$\frac{[\mathrm{PbPa}]_{\mathrm{crys}}}{[\mathrm{PbPa}]} = a - ae^{-(t-t_i)/\tau} \tag{4}$$

was fit to the concentration curves of crystalline PbPa without constraints using the `NonlinearModelFit` function in Mathematica, excluding data points before the onset of crystallization. For comparison, a second-order kinetics model

$$\frac{[\mathrm{PbPa}]_{\mathrm{crys}}}{[\mathrm{PbPa}]} = a - \frac{a}{1 + (t - t_{\mathrm{i}})/\tau} \tag{5}$$

was also fit on the crystalline PbPa concentration curves corresponding to 60 and 120 min heating time. All fits converged neatly.

2 Linear correlation between diamond phonon signal and temperature

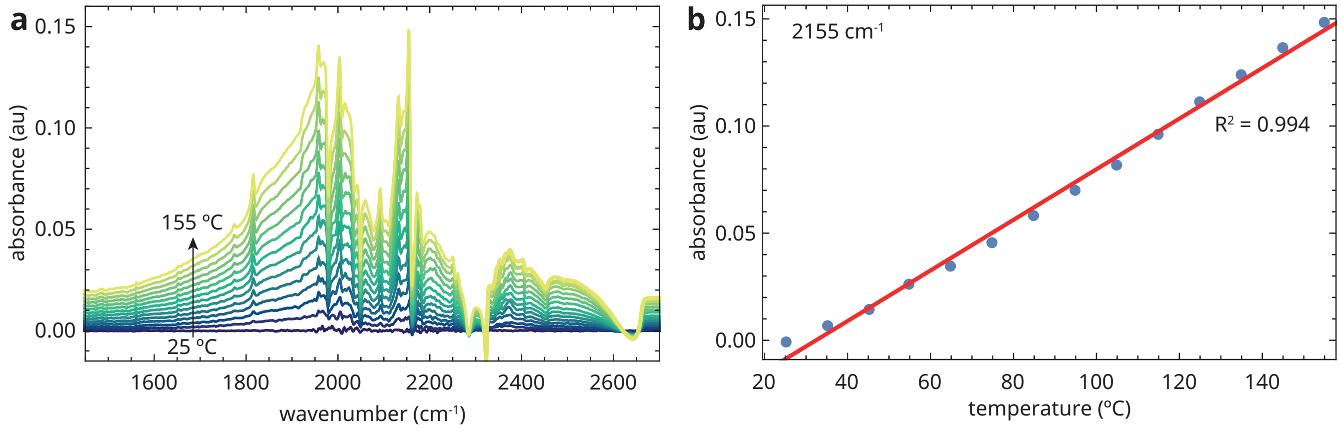

Figure S1 (a) Series of ATR-FTIR spectra recorded without a sample at 25–155 °C with a background at 25 °C, showing the temperature-dependent changes in the phonon signal of the ATR diamond. (b) Linear regression of the intensity at 2155 cm^{-1}, demonstrating that the background signal is approximately linear with temperature. Therefore, the diamond background signal can be used as a 'thermometer' for sample temperature during temperature-dependent ATR-FTIR experiments.

3 Long-term ZnPa crystallization processes in LO

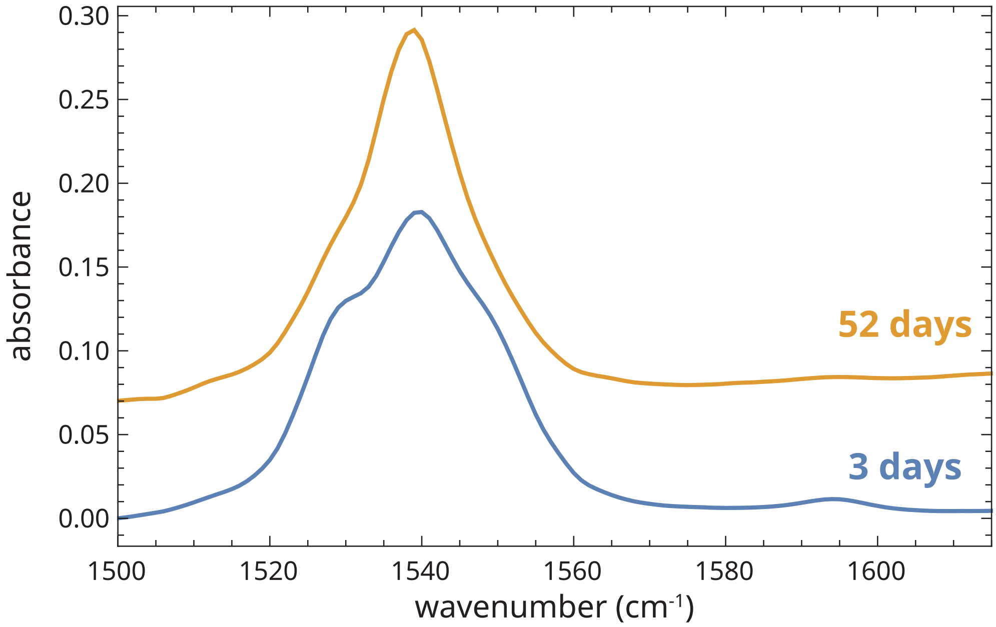

Figure S2 ATR-FTIR spectra of a mixture of ZnPa in LO measured 3 and 52 days after heating for 60 min at 150 °C.

4 ATR-FTIR spectra of pure ZnPa crystallization

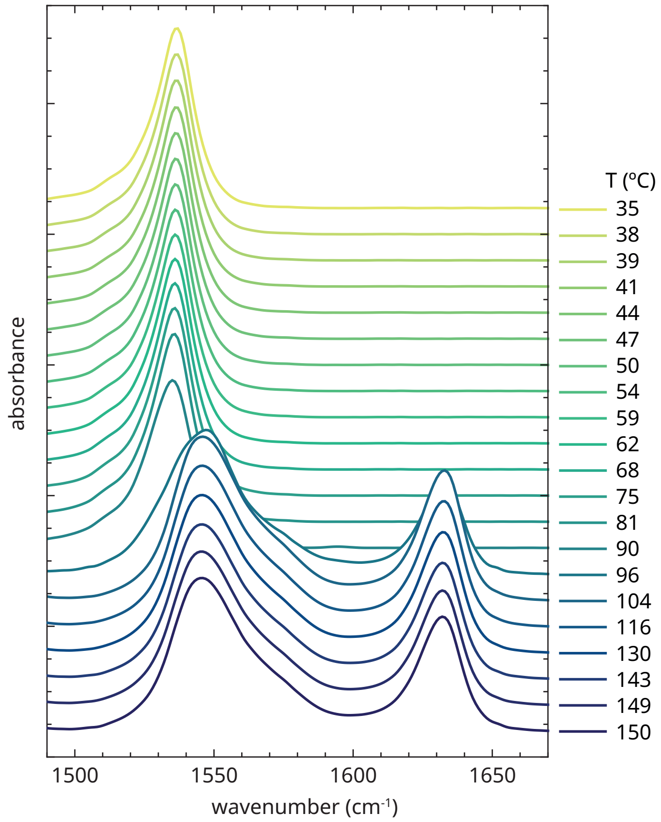

Figure S3 ATR-FTIR spectra of ZnPa recorded during cooling from 150 °C to room temperature, showing a rapid and direct conversion from the chain complex to crystalline ZnPa in the type B polymorph.

5 Comparison of first and second order kinetics for ZnPa crystallization

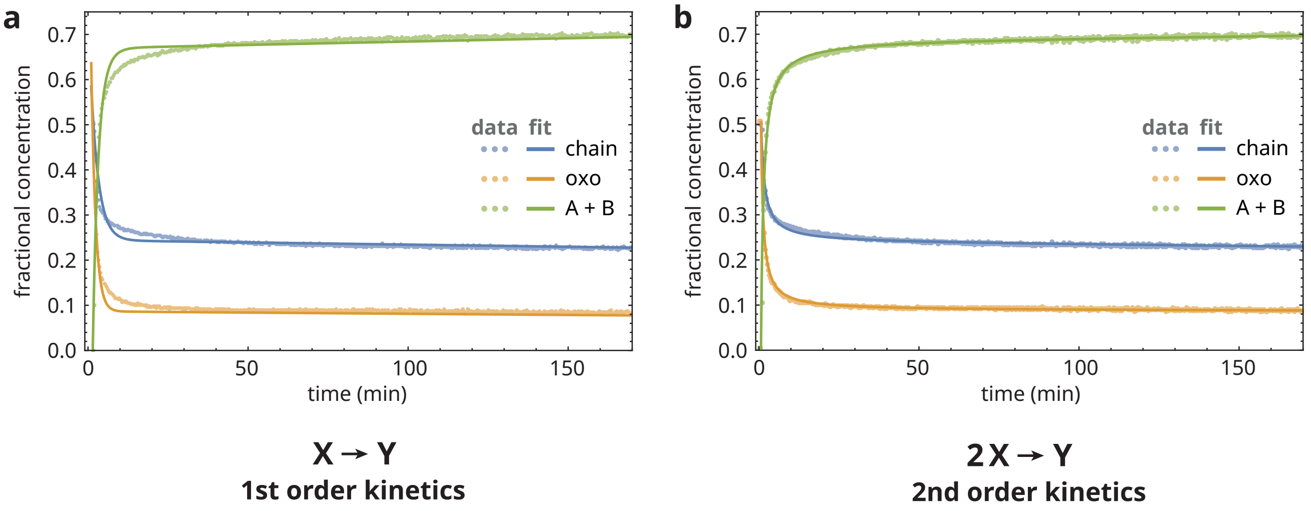

Figure S4 Comparison between (a) first order and (b) second order kinetics for the crystallization of ZnPa in LO heated at 150 °C for 15 min.

6 Kinetics of crystalline polymorph conversion of ZnPa in LO

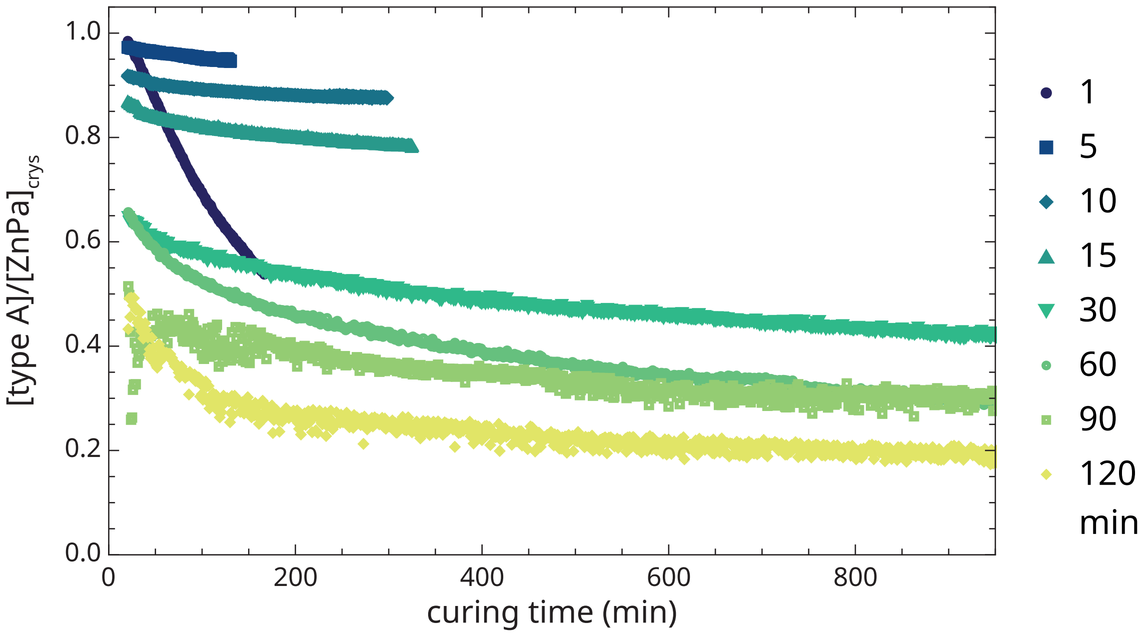

Figure S5 The fraction of crystalline ZnPa in the type A polymorph over time, for various degrees of matrix polymerization expressed as heating times at 150 °C. Apart from the mixture heated for just 1 min, most curves have a similar slope, suggesting that the type A → type B conversion process is largely independent of the level of polymerization or chemical properties of the oil matrix.

7 Comparison of first and second order kinetics for PbPa crystallization

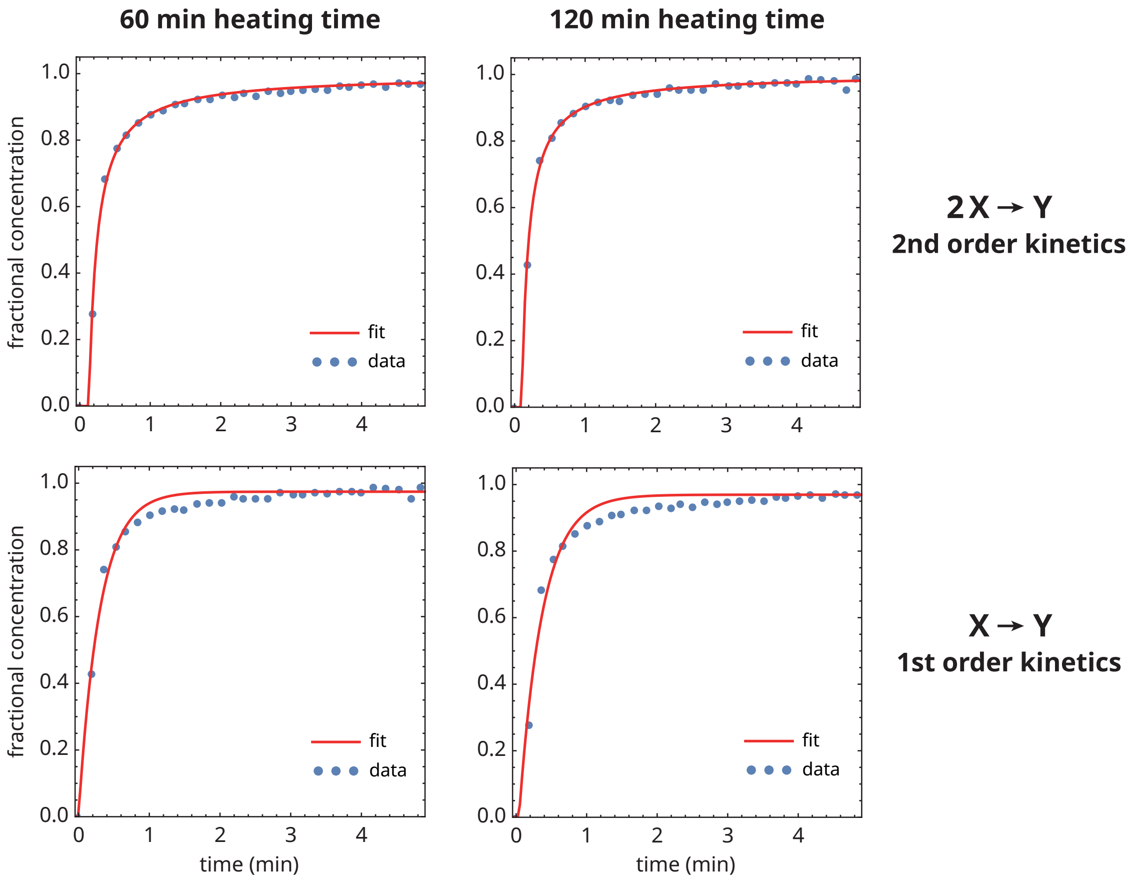

Figure S6 Comparison between first order and second order kinetics for the crystallization of PbPa in LO heated at 150 °C for 60 min and 120 min.
